# RNA 5-Methylcytosine Modification: Regulatory Molecules, Biological Functions, and Human Diseases

**DOI:** 10.1093/gpbjnl/qzae063

**Published:** 2024-09-28

**Authors:** Yanfang Lu, Liu Yang, Qi Feng, Yong Liu, Xiaohui Sun, Dongwei Liu, Long Qiao, Zhangsuo Liu

**Affiliations:** Department of Integrated Traditional and Western Nephrology, The First Affiliated Hospital of Zhengzhou University, Zhengzhou 450052, China; Research Institute of Nephrology, Zhengzhou University, Zhengzhou 450052, China; Henan Province Research Center for Kidney Disease, Zhengzhou 450052, China; Key Laboratory of Precision Diagnosis and Treatment for Chronic Kidney Disease in Henan Province, Zhengzhou 450052, China; Department of Integrated Traditional and Western Nephrology, The First Affiliated Hospital of Zhengzhou University, Zhengzhou 450052, China; Research Institute of Nephrology, Zhengzhou University, Zhengzhou 450052, China; Henan Province Research Center for Kidney Disease, Zhengzhou 450052, China; Key Laboratory of Precision Diagnosis and Treatment for Chronic Kidney Disease in Henan Province, Zhengzhou 450052, China; Department of Integrated Traditional and Western Nephrology, The First Affiliated Hospital of Zhengzhou University, Zhengzhou 450052, China; Research Institute of Nephrology, Zhengzhou University, Zhengzhou 450052, China; Henan Province Research Center for Kidney Disease, Zhengzhou 450052, China; Key Laboratory of Precision Diagnosis and Treatment for Chronic Kidney Disease in Henan Province, Zhengzhou 450052, China; Department of Integrated Traditional and Western Nephrology, The First Affiliated Hospital of Zhengzhou University, Zhengzhou 450052, China; Research Institute of Nephrology, Zhengzhou University, Zhengzhou 450052, China; Henan Province Research Center for Kidney Disease, Zhengzhou 450052, China; Key Laboratory of Precision Diagnosis and Treatment for Chronic Kidney Disease in Henan Province, Zhengzhou 450052, China; Department of Integrated Traditional and Western Nephrology, The First Affiliated Hospital of Zhengzhou University, Zhengzhou 450052, China; Research Institute of Nephrology, Zhengzhou University, Zhengzhou 450052, China; Henan Province Research Center for Kidney Disease, Zhengzhou 450052, China; Key Laboratory of Precision Diagnosis and Treatment for Chronic Kidney Disease in Henan Province, Zhengzhou 450052, China; Department of Integrated Traditional and Western Nephrology, The First Affiliated Hospital of Zhengzhou University, Zhengzhou 450052, China; Research Institute of Nephrology, Zhengzhou University, Zhengzhou 450052, China; Henan Province Research Center for Kidney Disease, Zhengzhou 450052, China; Key Laboratory of Precision Diagnosis and Treatment for Chronic Kidney Disease in Henan Province, Zhengzhou 450052, China; Department of Obstetrics and Gynecology, The First Affiliated Hospital of Zhengzhou University, Zhengzhou 450052, China; Department of Integrated Traditional and Western Nephrology, The First Affiliated Hospital of Zhengzhou University, Zhengzhou 450052, China; Research Institute of Nephrology, Zhengzhou University, Zhengzhou 450052, China; Henan Province Research Center for Kidney Disease, Zhengzhou 450052, China; Key Laboratory of Precision Diagnosis and Treatment for Chronic Kidney Disease in Henan Province, Zhengzhou 450052, China

**Keywords:** RNA methylation, m^5^C, m^5^C regulator, Biological process, Human disease

## Abstract

RNA methylation modifications influence gene expression, and disruptions of these processes are often associated with various human diseases. The common RNA methylation modification 5-methylcytosine (m^5^C), which is dynamically regulated by writers, erasers, and readers, widely occurs in transfer RNAs (tRNAs), messenger RNAs (mRNAs), ribosomal RNAs (rRNAs), enhancer RNAs (eRNAs), and other non-coding RNAs (ncRNAs). RNA m^5^C modification regulates metabolism, stability, nuclear export, and translation of RNA molecules. An increasing number of studies have revealed the critical roles of the m^5^C RNA modification and its regulators in the development, diagnosis, prognosis, and treatment of various human diseases. In this review, we summarized the recent studies on RNA m^5^C modification and discussed the advances in its detection methodologies, distribution, and regulators. Furthermore, we addressed the significance of RNAs modified with m^5^C marks in essential biological processes as well as in the development of various human disorders, from neurological diseases to cancers. This review provides a new perspective on the diagnosis, treatment, and monitoring of human diseases by elucidating the complex regulatory network of the epigenetic m^5^C modification.

## Introduction

According to the MODOMICS database of RNA modifications updated in 2023 [1], over 170 RNA modifications have been discovered. *N*^6^-methyladenosine (m^6^A) is the most common and widespread RNA modification in the majority of eukaryotes which accounts for approximately 1% of all adenine nucleotides. Cytosine methylated at carbon 5 with the formation of 5-methylcytosine (m^5^C), which was first identified in 1958 in *Escherichia coli* [2], is another RNA modification that has received much attention. Similar to m^6^A, m^5^C modification is reversible and can be influenced by proteins known as writers, readers, and erasers. Although m^5^C modification is not as abundant as m^6^A modification, which accounts for approximately 1% of all adenine nucleotides, m^5^C modification comprises 0.02%–0.09% of all cytosine nucleotides [3]. Recent studies have revealed that m^5^C is crucial for expression, alternative splicing, transport, stability, and translation of RNAs, and dysregulation of all these processes may cause various human diseases.

Owing to technical limitations, previous approaches for detecting m^5^C required substantial amounts of RNAs; hence, methylated sites were reliably detected only in highly abundant RNAs, such as transfer RNAs (tRNAs) and ribosomal RNAs (rRNAs). Nano-flow liquid chromatography coupled with triple tandem mass spectrometry (nLC-MS^3^) can quantitatively detect the total amount of m^5^C markers, but it cannot indicate the exact methylation sites in an RNA molecule [4]. Next-generation sequencing (NGS) technologies, such as m^5^C methylated RNA immunoprecipitation-sequencing (MeRIP-seq) [5], methylation individual-nucleotide-resolution cross-linking and immunoprecipitation with sequencing (miCLIP-seq) [6], RNA bisulfite sequencing (RNA-BisSeq) [7], and Aza-immunoprecipitation with sequencing (Aza-IP-seq) [8], facilitate the recognition of the precise locations of many m^5^C-modified nucleotides. However, many current approaches for detecting RNA changes have high error rates, low specificity, and poor repeatability. These sequencing technologies have been comprehensively described in previous articles [9]. Single-molecule sequencing, developed by Oxford Nanopore Technologies (ONT) in 2018, represents a further advancement in precision through direct RNA sequencing, eliminating the need for reverse transcription or amplification steps [9,10]. The results indicate that single-molecule sequencing directly identifies RNA changes, providing a new way of investigating epitranscriptomic alterations. Nevertheless, due to bioinformatic challenges, accurate detection of RNA m^5^C modification remains challenging. Therefore, there is a great need for new detection technologies in m^5^C methylation research.

**Figure 1:**
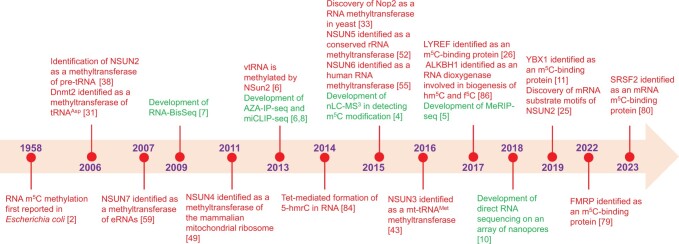
Key milestones in the discovery of RNA m^5^C methylation modifications Red represents the discovery of important m^5^C methylation-related regulatory molecules and green represents the development of m^5^C detection technologies. m^5^C, 5-methylcytosine; tRNA, transfer RNA; mRNA, messenger RNA; mt, mitochondrial; eRNA, enhancer RNA; ncRNA, non-coding RNA; lncRNA, long non-coding RNA; vtRNA, vault RNA; nLC-MS^3^: nano-flow liquid chromatography coupled with triple tandem mass spectrometry; MeRIP-seq, methylated RNA immunoprecipitation-sequencing; miCLIP-seq, methylation individual-nucleotide-resolution cross-linking and immunoprecipitation with sequencing; RNA-BisSeq, RNA bisulfite sequencing; Aza-IP-seq, Aza-immunoprecipitation with sequencing; hm^5^C, 5-hydroxymethylcytosine; f^5^C, 5-formylcytosine; 5-hmrC, 5-hydroxymethylcytidine.

Although the m^5^C methylation was first identified many years ago, an explosive phase of research on this epigenetic modification has only recently begun. According to a review of the PubMed database, the development of techniques to identify m^5^C sites using NGS has been the primary driver for the boom in m^5^C modification studies. Many milestones have been achieved, such as the discovery of m^5^C methylation-associated proteins and the development of m^5^C methylation-associated sequencing technologies. These milestone events stimulated further studies into m^5^C modification. The landmark discoveries related to RNA m^5^C modification research in the decades following its formal description in 1958 are shown in **Figure 1**.

RNA m^5^C methylation is detected in various animal tissues, such as the bladder [11], liver [12], lung [13], and plants such as *Arabidopsis thaliana* [5], as well as in different microorganisms (including bacteria, viruses, archaea, and yeast) [14]. A summary of current research advances in the field of m^5^C modification and its roles in human disorders is necessary because of the widespread distribution of this epigenetic marker. In this review, we explored the regulators of m^5^C modification involved in its emergence, distribution in various RNAs, and the impact of this epigenetic tag on RNA functions. We focused on the important mechanisms affected by RNA m^5^C modification in crucial biological processes, such as embryonic development, cell fate determination, and cancer progression. Additionally, we discussed the roles of methylation with m^5^C marks under various human pathological conditions and its potential applications in the treatment of human diseases, ranging from neurological disorders to cancers.

## Distribution of RNA m^5^C methylation

RNA m^5^C methylation has been found in tRNAs, rRNAs, messenger RNAs (mRNAs), and various non-coding RNAs (ncRNAs), including long non-coding RNAs (lncRNAs) [15–17] and circular RNAs [18]. Because of the requirement of high RNA amounts for analysis, the two most abundant groups of RNAs (tRNAs and rRNAs) account for the greatest majority of m^5^C methylation. RNA m^5^C sites commonly exist in the variable regions and anticodon loops of tRNA molecules. These sites stabilize tRNA secondary structure, while also regulating codon identification and tRNA aminoacylation. In eukaryotic tRNAs, m^5^C residues cluster around the intersection of the variable area and TΨC stem. In both eukaryotic and archaeal tRNAs, m^5^C modifications often occur at positions C48 and C49. Higher eukaryotes have an additional m^5^C residue at position C72 of the tRNA acceptor stem [19]. The m^5^C methylation, which primarily affects protein synthesis, is found in nuclear, cytoplasmic, and mitochondrial rRNAs. In the nucleolus, NSUN1 and NSUN5 methylate the 25S rRNA in domain V [20]. In the cytoplasm, NSUN1 is responsible for the methylation of 25S rRNA [21], whereas in the mitochondria, NSUN4 is responsible for the methylation of cytosine 911 in the 12S rRNA [22]. However, few RNA modifications have been discovered in less abundant RNAs including microRNAs, small nuclear RNAs, and small nucleolar RNAs [23,24].

Because of the advances in NGS technologies, mRNA m^5^C methylation has been detected more frequently, gradually becoming a new, intensively researched topic. To briefly summarize, there are typically several hundred mRNA m^5^C sites in a given adult tissue in mammals. mRNA m^5^C is prominently enriched in maternal mRNAs. Tumor cells generally exhibit a higher density of mRNA m^5^C compared to normal cells [25,26]. In contrast to the consensus RRACH motif for m^6^A detected using RNA-BisSeq in most species, the sequence feature for m^5^C shows enrichment only around the CG-rich region in distinct species [27,28]. In 2013, Edelheit et al. first identified the consensus motif AUCGANGU in prokaryotic mRNAs using bisulfite treatment combined with m^5^C-specific RNA immunoprecipitation [14]. Squires et al. demonstrated that in human mRNAs, m^5^C is commonly found in both the coding regions and untranslated regions (UTRs) [29]. Amort et al. have shown that poly(A) RNAs exhibit distinct m^5^C modification profiles, with mouse embryonic stem cells displaying a greater diversity of methylated mRNAs compared to the brain, although both cell types demonstrate an enrichment of these modifications in 3′-UTRs. In contrast, there is a greater increase in the m^5^C frequency in nuclear poly(A) RNA than in total poly(A) fractions, which may be related to RNA splicing or transcript degradation processes [15].

## Regulators of RNA m^5^C methylation and their biological functions in mammals

### Methyltransferases (writers)

**Figure 2:**
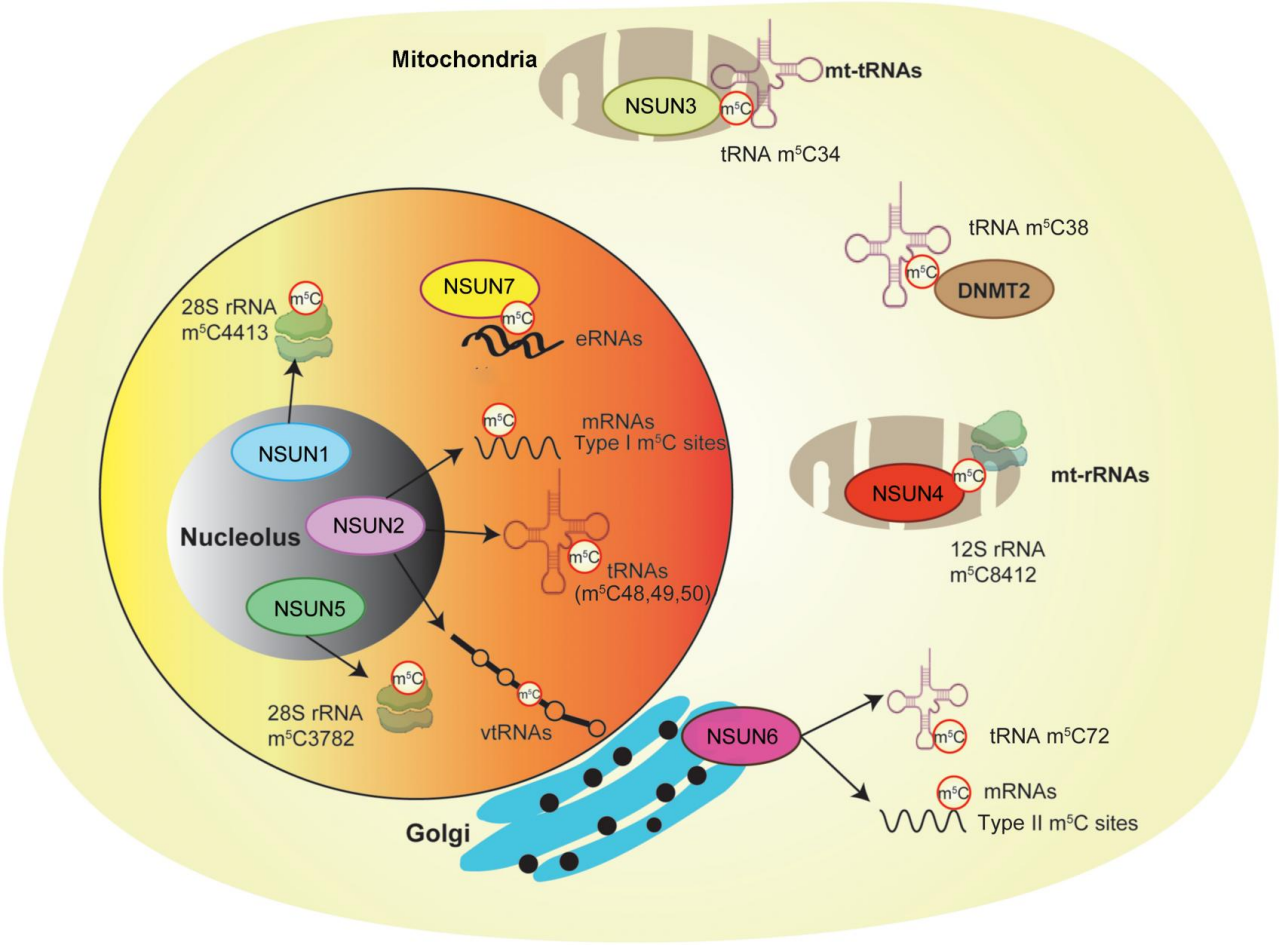
Sequence and structural features of the RNA substrates specific to each enzyme rRNA, ribosomal RNA.

The common characteristics of RNA m^5^C methyltransferases include a catalytic domain with a structural core of approximately 270 amino acids and an *S*-adenosyl methionine-binding site [30]. DNMT2 and the NSUN family members (NSUN1–NSUN7) catalyze the emergence of m^5^C modification in mammals [31,32]. Although NSUN1, NSUN2, and NSUN5 are present in all eukaryotes, the remaining NSUN family proteins are exclusively found in higher eukaryotes. For the nucleophilic assault on carbon 6 of the target cytosine in RNA, NSUN family enzymes employ cysteine from amino acid motif VI. **Figure 2** illustrates the sequence and structural features of the RNA substrate specific to each enzyme.

#### The NSUN family members

NSUN1 (also known as NOP2, NOL1, or p120) is a nucleolar protein that acts as an oncogene; its expression is dysregulated in various cancers. The NOP2/NSUN1 homolog in *Saccharomyces cerevisiae* catalyzes m^5^C deposition on the 25S rRNA at position C2870, which is close to the ribosome peptidyl transferase center [33]. NSUN1, an rRNA methyltransferase, methylates cytosine at position C2982 of the 26S rRNA, which affects the health span and oogenesis of *Caenorhabditis elegans* [34]. Data obtained using miCLIP-seq in human cells showed that NSUN1 also catalyzes the methylation of cytosine at position C4447 of the 28S rRNA. It regulates ribosome biogenesis by binding to pre-rRNA transcripts and is responsible for regulating pre-RNA processing through non-catalytic complex formation with box C/D of small nucleolar RNAs [35].

NSUN2 is an m^5^C RNA methyltransferase with a broad substrate specificity that targeting the majority of tRNAs and ncRNAs, and it has also been recently identified as a methylator of mRNAs as well [6,11,12,27,36–38]. NSUN2 methylates type I m^5^C sites, which have a downstream G-rich triplet motif and are computationally anticipated to be situated at the 5′ end of putative hairpin structures. NSUN2 is predominantly localized in the nucleus. In 2019, Shinoda et al. demonstrated the methylation of cytosines by NSUN2 in mitochondrial tRNAs at positions C48, C49, and C50 [39,40]. Vault RNAs (vtRNAs) are ncRNAs incapable of encoding proteins [41]. However, vtRNAs have been implicated in multiple cellular functions. In addition to methylating mRNAs and tRNAs, NSUN2 has also been reported to mediate RNA m^5^C methylation in vtRNA1.1/1.3 to regulate epidermal differentiation [42].

NSUN3 is mainly located in the mitochondria, where it modifies the wobble position of mitochondrial methionine tRNA (mt-tRNA^Met^) to expand codon recognition in mitochondrial translation [43,44]. NSUN3 plays an essential role in mitochondrial translation, specifically methylating mitochondrial tRNA 5-formylcytidine modification [45]. Mutations in the human *NSUN3* gene have been associated with mitochondrial diseases. Previous studies have demonstrated that NSUN3 regulates embryonic stem cell differentiation by influencing mitochondrial activity [46]. Moreover, *Nsun3* knockout in mice results in embryonic lethality while mice with heart-specific *Nsun3* knockout in adulthood exhibit enlarged and fragmented cristae [45].

NSUN4 interacts with MTERF4 to specifically methylate an unknown residue in 16S rRNA within mitochondria both *in vitro* and *in vivo* [47–49]. RNA-BisSeq has shown that in mouse heart mitochondrial rRNAs, NSUN4 is responsible for the methylation of cytosine 911 in the 12S rRNA of the small subunit [50]. In 2020, Navarro et al. reported that NSUN4 acts as a dual multisite-specific rRNA/tRNA methyltransferase in *C*. *elegans* mitochondria, influencing nematode adaptation to higher temperatures [22].

NSUN5, a conserved rRNA methyltransferase, is responsible for the methylation at position C2278 of the 25S rRNA in yeast as well as at position C3381 of the 26S rRNA in worms, which modulates the lifespan of the organisms [51,52]. Furthermore, NSUN5 methylates the conserved human and mouse 28S rRNAs at positions C3782 and C3438, respectively [20,53,54].

Previous studies have shown that NSUN6 is widely expressed, with the highest levels in the testis and lowest levels in the blood. This methyltransferase methylates both tRNAs and mRNAs [42,44]. NSUN6-specific methylated sites are enriched in the 3′-UTR within the consensus sequence motif CTCCA [44]. In humans, NSUN6 acts as a tRNA methyltransferase, with threonine tRNA (tRNA^Thr^) and cysteinyl tRNA (tRNA^Cys^) being RNA substrates, and C72 at the 3′ end of the tRNA acceptor stem as the target nucleotide [55]. Previous studies have also revealed that NSUN6 is an mRNA m^5^C methyltransferase that targets type II m^5^C sites containing a downstream UCCA motif [56–58]. NSUN6 and NSUN2 work on distinct subsets of mRNA m^5^C sites and together are responsible for nearly all m^5^C modifications in mRNAs, as shown by mRNA BisSeq [56].

NSUN7 was initially discovered as an enhancer RNA (eRNA) m^5^C methylation transferase [59]. In liver cell model systems, NSUN7 can methylate *Pfkl*-, *Sirt5*-, *Idh3b*-, and *Hmox2*-associated eRNAs, influencing their stability, as confirmed by the Aza-IP-seq, RNA immunoprecipitation PCR (RIP-qPCR), and Methylamp RNA bisulfite conversion methods [59].

#### DNMT2/TRDMT1

DNMT2 is a DNA methyltransferase homolog that specifically methylates aspartic acid tRNA (tRNA^Asp^) [31]. In 2006, DNMT2 was shown to methylate C38 in the anticodon loop of tRNA^Asp(GUC)^ in mice, *A*. *thaliana*, and *Drosophila melanogaster* [31]. In humans, it also acts as a tRNA methyltransferase that methylates cytosines in the anticodon loops of tRNA^Asp(GUC)^, tRNA^Gly(GCC)^, tRNA^Glu(CUC)^, and tRNA^Val(AAC)^ [60,61]. Furthermore, Li et al. characterized the substrate properties and recognition mechanisms of DNMT2/TRDMT1. They demonstrated that tRNA^Gly(GCC)^ is the preferential substrate of human DNMT2/TRDMT1 *in vitro*. This tRNA m^5^C modification promotes tRNA stability and translation of a specific subset of genes [62].

### Binding proteins (readers)

#### ALYREF

ALYREF contains a canonical RNA-binding motif and mainly binds the 5′- and 3′-regions involved in mRNA export [63]. ALYREF has the ability to recognize m^5^C sites and acts as an m^5^C nuclear reader [28]. It can mediate the transport of m^5^C-modified RNAs from the cytoplasm to the nucleus and maintain their stability. ALYREF has been implicated in the development of several malignancies through m^5^C modification. Several m^5^C-modified mRNAs, including *PFAS*, *RABL6/TK1*, *LRRC8A*, *Myc*, *PKM2*, and *YAP1*, have been identified as potential ALYREF targets in cancer [64–66]. Moreover, ALYREF has been associated with viral replication; for example, it promotes retrovirus replication in an RNA m^5^C modification-dependent manner [67]. By localizing m^5^C-modified *YBX2* and *SMO* mRNAs and exporting them from the nucleus to the cytoplasm, ALYREF increases the production of YBX2 and SMO proteins, inhibits adipogenesis, and promotes myogenesis [68].

#### YBX1

YBX1 is a multifunctional protein containing an evolutionarily conserved cold-shock domain (CSD). YBX1 belongs to the RNA-binding protein (RBP) family and is involved in both transcription and translation as a splicing factor [69]. Additionally, YBX1 can enhance the stability and expression of gene transcripts by specifically recognizing the m^5^C modification [11,70–72]. YBX1 plays an important role in multiple diseases (including cancer) and maternal-to-zygotic transition (MZT), in an m^5^C-dependent manner. YBX1 has been identified to target several genes, including *TSPAN13* [71], *PFKFB4* [73], *TIAM2* [74], *QSOX1* [75], *ORAI2* [76], and *HDGF* [11].

#### YBX2

YBX2 is a recently identified novel m^5^C reader protein in the cytoplasm. It is specifically more abundant in mammalian testis than in other tissues and shares a conserved CSD with the known RNA m^5^C reader YBX1 [77]. Further structural analysis has revealed that W100 is the key residue responsible for recognizing m^5^C-modified RNAs. YBX2 has the capacity to promote liquid–liquid phase separation of m^5^C-labeled RNA, both *in vivo* and *in vitro* [78].

#### Fragile X mental retardation protein

Fragile X mental retardation protein (FMRP) is a cytoplasmic RBP that regulates protein translation. In 2022, it was discovered that FMRP serves as an m^5^C reader, acting as a coordinator between the m^5^C writer TRDMT1 and eraser ten-eleven translocation 1 (TET1), and this coordination facilitates mRNA-dependent repair and cell survival in cancer [79]. This study unveiled FMRP as a novel addition to the family of RNA recognition proteins.

#### Serine/arginine-rich splicing factor 2

Serine/arginine-rich splicing factor 2 (SRSF2) is a member of serine/arginine-rich (SR) proteins which are RBPs playing important role in RNA splicing. SRSF2 is a multifunctional protein that modulates RNA splicing, transcriptional elongation, and RNA stability. In 2023, Ma et al. first reported SRSF2 as a m^5^C-binding protein in which mutation P95H is associated with poor outcome in leukemia. SRSF2 binds preferentially to m^5^C-modified RNAs with specificity in the C(m^5^C)GG context [80]. This was the first reported a previously unrecognized reader of m^5^C on mRNAs.

### Demethylases (erasers)

#### The TET family members

The TET family members include TET1, TET2, and TET3, which are also known as DNA demethylases [81]. In 2014, using LC-MS/MS, Fu et al. demonstrated that TETs oxidize m^5^C to 5-hydroxymethylcytosine (hm^5^C) in the RNAs of HEK293T cells [82]. In 2016, Delatte et al. mapped the transcriptome-wide distribution of hydroxymethylcytidine (hmrC) in RNAs in *Drosophila* cells and observed that the *Drosophila* brain contained high levels of TET and hydroxymethylated RNAs. Additionally, they found that TET-deficient fruit flies exhibited impaired brain development and reduced level of RNA hydroxymethylation [83]. The TET family has an impact on tRNA methylation, which subsequently influences tRNA translation. TET-mediated RNA hydroxymethylation reduces the stability of crucial pluripotency-promoting transcripts, such as *Eed* and *Jarid2* [84].

#### ALKBH1

ALKBH1 is a dioxygenase responsible for the sequential conversion of m^5^C to hm^5^C and 5-formylcytosine (f^5^C) at position C34 of cytoplasmic and mitochondrial tRNAs. This enzyme activity has been shown to affect mitochondrial activity by reducing translation and oxygen consumption [85,86]. In this context, the conversion of m^5^C into hm^5^C reduces the overall m^5^C modification level. Therefore, both TETs and ALKBH1 are considered as RNA m^5^C methylation erasers.

The chemical RNA m^5^C methylation is a dynamically reversible process mediated by several enzymes and relevant methylation recognition proteins as mentioned above. **Table 1** summarizes the distribution of different regulators of RNA m^5^C methylation and their targets.

**Table 1:** Human m^5^C methyltransferases and their RNA targets

Category	Protein	**Subcellular** **localization**	Target RNA	Modified site installed	Refs.
Writer (methyltransferase)	NSUN1	Nucleolus	28S rRNA	m^5^C4447	[35]
	NSUN2	Nucleus/nucleolus	Pre-tRNA^Leu(CAA)^	m^5^C34	[38]
			tRNA^Ala(AGC/CGC/UGC)^, tRNA^His(GUG)^, tRNA^Ile(AAU)^, tRNA^Leu(CAA/AAG/CAG/UAA/UAG)^, tRNA^Lys(CUU)^, tRNA^Met(CAU)^, tRNA^Ser(AGA/CGA/GCU/UGA)^, tRNA^Thr(CGT/UGU)^, tRNA^Tyr(GUA)^	m^5^C48	[36,62]
			tRNA^Asp(GUC)^, tRNA^Gln(CUG/UUG)^, tRNA^Lys(UUU)^, tRNA^Phe(GAA)^, tRNA^Thr(AGU)^, tRNA^Val(AAC/CAC/UAC)^	m^5^C48,49	[36,62]
			tRNA^Glu(CUC/UUC)^, tRNA^Gly(CCC/GCC/UCC)^, tRNA^Pro(AGG/CGG/UGG)^	m^5^C48,49,50	[36,62]
			vtRNA1.1	m^5^C69	[6]
			vtRNA1.2	m^5^C27,59	[6]
			vtRNA1.3	m^5^C15,27,59	[6]
			mRNAs	Type I m^5^C sites	[28,37]
	NSUN3	Mitochondria	mt-tRNA^Met^	m^5^C34	[44]
	NSUN4	Mitochondria	mt-12S rRNA	m^5^C8412	[50]
	NSUN5	Nucleolus	28S rRNA	m^5^C3782	[20,54]
	NSUN6	Cytoplasm/Golgi	tRNA^Cys/Thr^ mRNAs	m^5^C72 Type II m^5^C sites	[55,58] [56,57]
	NSUN7	Nucleus	eRNAs	Various	[59]
	DNMT2	Cytoplasm/nucleus	tRNA^Asp(GUC)^, tRNA^Gly(GCC)^, tRNA^Val(AAC)^	m^5^C38	[31,61]
Reader (binding protein)	ALYREF	Nucleus	*BX2* and* SMO* mRNAs	NA	[68]
	YBX1	Cytoplasm/nucleus/ cytoplasmic granule	Various	NA	[11,72]
	YBX2	Cytoplasm	Various	NA	[78]
	FMRP SRSF2	Cytoplasm Nucleus	mRNAs at DSBs mRNAs	NA C(m^5^C)GG	[79] [80]
Eraser (demethylase)	TET1/TET2/TET3	Nucleus	m^5^C to 5-hmrC	NA	[82]
	TET2	Nucleus	m^5^C to hm^5^C on tRNAs	NA	[81]
	ALKBH1	Nucleus/cytoplasm/mitochondria	tRNA^Leu^ and mt-tRNA^Met^	m^5^C34	[86]

*Note*: m^5^C, 5-methylcytosine; tRNA, transfer RNA; mRNA, messenger RNA; rRNA, ribosomal RNA; eRNA, enhancer RNA; vtRNA, vault RNA; mt, mitochondrial; DSB, double-strand break; 5-hmrC, 5-hydroxymethylcytidine; hm^5^C, 5-hydroxymethylcytosine.

## RNA m^5^C methylation in crucial biological processes

**Figure 3:**
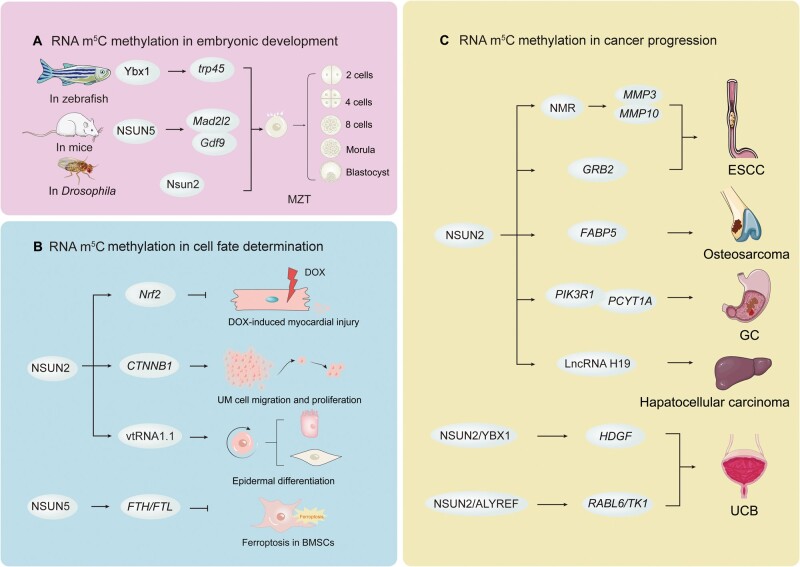
Roles of RNA m^5^C methylation in crucial biological processes Roles of RNA m^5^C methylation in embryonic development (**A**), cell fate determination (**B**), and cancer progression (**C**). MZT, maternal-to-zygotic transition; ESCC, esophageal squamous cell carcinoma; UCB, urothelial carcinoma of the bladder; BMSC, bone marrow mesenchymal stem cell; GC, gastric cancer; UM, uveal melanoma; DOX, doxorubicin.

Dysregulation of RNA modification regulators affects various biological activities. Below, we summarized the mechanisms by which RNA m^5^C methylation plays important roles in embryonic development, cell fate determination, and cancer progression. **Figure 3** shows the roles of the m^5^C epigenetic alteration in multiple biological processes.

### RNA m^5^C methylation in embryonic development

MZT is a critical process that involves the fusion of two distinct gametes (sperm and oocyte), resulting in a totipotent zygote state. In 2019, Yang et al. demonstrated that in zebrafish, RNA m^5^C methylation regulates the stabilization of maternal mRNAs during MZT through the YBX1/PABPC1A–TRP45 signaling pathway [72]. In 2022, Liu et al reported that *Drosophila*, a invertebrate animal whose embryos lack maternal mRNA m^5^C due to the knockout of *Nsun2*, can also experience cell cycle delays and fail to promptly initiate MZT [87].

Moreover, NSUN5 deficiency decreased m^5^C levels in exons and 3′-UTRs, which altered the efficiency of *Mad2l2* and *Gdf9* translation in the mouse ovary. NSUN5 deficiency impaired follicular genesis and development, indicating that m^5^C-regulated stability of maternal *Nsun5* mRNA is required for MZT [88]. These studies highlight the critical role of mRNA m^5^C methylation in the early development of invertebrates and vertebrates.

### RNA m^5^C methylation in cell fate determination

RNA m^5^C methylation plays a crucial role in regulating various aspects of cell fate decisions, including proliferation, differentiation, motility, apoptosis, and ferroptosis. For example, NSUN2-mediated m^5^C methylation of the *CNTTB1* mRNA has been shown to modulate uveal melanoma cell proliferation and migration through the induction of cell cycle G1 arrest [89]. RNA m^5^C methylation is also involved in cell differentiation. In 2019, Sajini et al. reported that deletion of *NSUN2* influenced the methylation of vtRNA1.1, and the unmethylated vtRNA1.1 was recognized by SRSF2 to regulate epidermal differentiation [42]. NSUN2-mediated m^5^C methylation in tRNAs is believed to be essential for the differentiation and motility of epidermal and neuroepithelial stem cells [90,91]. Owing to the critical role of NSUN2 in facilitating neural stem cell differentiation, NSUN2 deficiency has been associated with several developmental diseases.

RNA m^5^C methylation regulates cell death (apoptosis and ferroptosis). Apoptosis is an active physiological process of cell death under certain physiological or pathological conditions, controlled by intrinsic genetic mechanisms. NSUN2 can alleviate doxorubicin (DOX)-induced myocardial injury and apoptosis through NRF2-mediated antioxidant activity in an m^5^C-dependent manner [92]. Liu et al. reported that NSUN5 inhibited ferroptosis by targeting ferritin heavy and light chains and improved the survival of transplanted bone marrow-derived mesenchymal stem cells in an m^5^C-dependent manner [93].

### RNA m^5^C methylation in cancer progression

RNA m^5^C methylation has been extensively studied during cancer progression. NSUN2 is an important m^5^C writer that plays an important role in various cancers, including esophageal squamous cell carcinoma (ESCC), urothelial carcinoma of the bladder (UCB), gastric cancer (GC), hepatocellular carcinoma, and osteosarcoma. Mechanistically, NSUN2 promotes tumor development mainly through the methylation of related mRNAs and lncRNAs. It has been reported that tRNA m^5^C has a significant impact on the survival of tumor-initiating cells, as well as on tumor development and metastasis, and this effect is induced by *Nsun2* knockout [94]. *NSUN3*-deficient tumor switched to glycolysis and failed to metastasize, which is attributed to the modulation of mitochondrial m^5^C and f^5^C [95]. Figure 3C shows specific molecular mechanisms through which RNA m^5^C methylation promotes tumor development.

## RNA m^5^C methylation in human diseases

### Hereditary diseases

In humans, mutations in *NSUN2* can lead to disorders accompanied by mental disability. In 2012, Lia Abbasi-Moheb et al. reported two nonsense mutations and one splicing mutation that caused a loss of *NSUN2* function in three independent consanguineous Iranian and Kurdish families. To further investigate the role of mutated *NSUN2*, the *NSUN2* ortholog CG6133 was deleted in *Drosophila*, which resulted in severe short-term memory abnormalities in mutant flies [96]. In the same year, Khan et al. identified the missense change c.2035G>A (p.Gly679Arg) within *NSUN2*. In a mouse model, transfection with such mutant *Nsun2* caused cognitive disturbances [97]. Martinez et al. first reported that mutations in *NSUN2* cause Dubowitz-like syndrome, which is characterized by a constellation of mild microcephaly, growth and mental retardation, eczema, and peculiar facies. These manifestations were likely caused by the lack of m^5^C modifications in tRNA^Asp(GTC)^ [98]. In 2014, Blancono et al. reported that the accumulation of 5′ tRNA fragments in the absence of NSUN2 reduced protein translation rates and activated stress pathways [36]. These studies demonstrate the importance of NSUN2-induced methylation of tRNAs for normal cognitive development. These studies also provide a basis for our understanding of NSUN2 functions and facilitate the diagnosis and treatment of NSUN2-related diseases.

#### Mitochondrial respiratory chain complex deficiency

Mitochondrial deficiency disorders are characterized by microcephaly, failure to thrive, recurrently elevated plasma lactate levels, muscle weakness, proximal exacerbated external ophthalmoplegia, and convergence nystagmus. Haute et al. reported that a mutation in *NSUN3* led to m^5^C deficiency in mt-tRNA^Met^ at position C34 (m^5^C34), which resulted in a lack of f^5^C at the same tRNA position, eventually leading to mitochondrial respiratory chain complex deficiency [44,99]. These results show that NSUN3 is essential for the efficient translation and activity of proteins in the mitochondria.

#### Williams–Beuren syndrome

The deletion of *NSUN5* is linked to Williams–Beuren syndrome (WBS), which is characterized by a unique cognitive profile. This profile includes relatively intact expressive language, difficulties with facial processing, and significant impairments in spatial recognition [100]. *NSUN5* deletion has been observed in approximately 95% of patients with WBS [101]. Spatial cognitive impairment has been observed in *Nsun5*-knockout mice. *Nsun5* deletion suppressed the activity of the *N*-methyl-D-aspartic acid (NMDA) type of glutamate receptors in neuronal cells, which could possibly be attributed to disrupted development and function of oligodendrocyte precursor cells. This resulted in deficits in NMDA receptor-dependent long-term potentiation and spatial cognitive abilities [102]. Haploinsufficiency of *NSUN5* in fibroblasts of WBS patients resulted in a partial loss of the m^5^C3782 modification in the 28S rRNA, which led to a reduction in total protein synthesis due to altered ribosomes [20].

#### Cardiac outflow tract disorders

*NSUN5* mutation is associated with the development of outflow tract (OFT) disorders. In a recent study conducted by Wang et al., four potential pathogenic mutations were found in the coding region of the *NSUN5* gene in 132 patients with tetralogy of Fallot and 2000 controls. Mechanistic studies have shown that NSUN5 is required for normal OFT morphogenesis and regulates the *Tpm1* gene as an m^5^C methyltransferase [103]. The detection of *NSUN5* mutation can facilitate the diagnosis of OFT [104].

### Cancers

Abnormal patterns of RNA methylation with m^5^C marks are found in many cancers, including ESCC, UCB, GC, pancreatic cancer, and hepatocellular carcinoma. Methylation of various mRNAs and lncRNAs has oncogenic and metastasis-promoting effects.

In ESCC, a lncRNA NMR, methylated by NSUN2, was found to be significantly up-regulated. This up-regulation was associated with tumor metastasis and drug resistance. NMR bound directly to the chromatin regulator BPTF, potentially promoting MMP3 and MMP10 expression through the ERK1/2 pathway by recruiting BPTF to chromatin. This suggests a mechanistic link between NMR and MMP3/MMP10 expression [105]. Another study reported that NSUN2-mediated methylation of RNAs with m^5^C marks promoted ESCC progression through LIN28B-dependent *GRB2* mRNA stabilization [106].

In UCB, NSUN2, along with YBX1, stabilizes mRNA of the oncogene *HDGF*, which mediates UCB pathogenesis in humans. This provides a therapeutic rationale for targeting the NSUN2/YBX1/m^5^C-HDGF signaling axis in UCB patients [11]. In addition, a recent study reported that NSUN2 and ALYREF facilitated UCB progression by influencing *RABL6*/*TK1* mRNA splicing and RNA stabilization [66]. NSUN2 was also abundantly expressed in GC and linked with poor prognosis in patients due to its promotion of the proliferation and metastasis of GC cells *in vitro* by targeting *PIK3R1* and PCYT1A [107]. In hepatocellular carcinoma, *NSUN2* acted as an oncogene by methylating lncRNA H19, which then bound to *G3BP1*, promoting its stabilization, thereby leading to carcinogenesis [108]. NSUN2 accelerated osteosarcoma development by increasing *FABP5* mRNA stability through m^5^C modification [109]. In addition, NSUN2 suppressed epithelial differentiation in pancreatic cancer through mRNA m^5^C modification [110]. NSUN2 was also involved in chemotherapy resistance in various tumors. For example, *cis*-expression quantitative trait loci (*cis*-eQTLs) in *NSUN2* promoted ESCC progression and radio-chemotherapy resistance through mRNA m^5^C modification [111].

NSUN6 regulated cell proliferation and was shown to be down-regulated in pancreatic cancer [112]. Awahet et al. discovered that elevated NSUN6 expression improved survival in glioblastoma and other malignancies. NSUN6 also regulated the sensitivity to chemotherapeutic drugs. For example, NSUN6 affected the response to temozolomide therapy through the m^5^C-mediated regulation of *NELFB* and *RPS6BK2* mRNA expression in glioblastoma [113].

The deletion of *DNMT2*/*TRDMT1* in cancer cells impaired the DOX-induced unfolded protein response and increased the susceptibility of cancer cells to endoplasmic reticulum stress-induced death [114]. Knocking out the *DNMT2*/*TRDMT1* gene in drug-treated glioblastoma cells led to a decrease in the number of apoptotic and senescent cells, a reduction in interleukin-8 levels and autophagy, and an increase in the number of necrotic cells, compared with those observed in drug-treated glioblastoma cells with unmodified levels of *DNMT2*/*TRDMT1* [115]. Moreover, redox homeostasis, proliferation-related pathways, and DNMT2/TRDMT1-based effects could be modulated as part of an anti-osteosarcoma strategy, reflecting the diverse phenotypic features of osteosarcoma cells [116]. DNMT2 has been implicated in the response of cancer cells to drugs.

In addition to the role of these methyltransferases in tumorigenesis and development, demethylation enzymes also play an important role in cancer. A recent study by Li et al. reported that TET2 could act as an m^5^C eraser to regulate leukemia stem cell homing and self-renewal through m^5^C-mediated *TSPAN13* mRNA stability. Mechanistically, TET2 deficiency causes m^5^C accumulation in *TSPAN13* mRNA; YBX1 selectively identifies the modification and improves the stability and expression of *TSPAN13* transcripts [71]. This finding suggests that TET2 plays an important role in acute myeloid leukemia as an mRNA m^5^C demethylase.

Recent studies using data mining from the Gene Expression Omnibus and The Cancer Genome Atlas databases have also demonstrated that m^5^C regulators could predict the prognosis of various cancers [117–124]. Although these findings can assist in the clinical diagnosis, treatment, and prognosis of cancer, the molecular mechanisms underlying the effects of these regulators on cancer development have not been thoroughly investigated. Therefore, there is an urgent need to study the functions of RNA m^5^C methylation in tumorigenesis, metastasis, and therapeutic interventions.

### Other diseases

#### Infertility

Recently, there have been several reports on the relationship between *NSUN7* mutation and infertility. Sperm motility defects and infertility have been observed in male mice with a mutation in *Nsun7* [125]. The chemically induced mutation of Ste5Jcs1 in *Nsun7* induced defects in the motility of sperm and infertility in male mice; however, the molecular mechanism of this phenotype remains unclear [125]. Researchers have found that deletion of adenine at position A11337 in exon 4 of *NSUN7* produced an abortive, shorter protein product and was linked to sperm motility problems in infertile men [126]. Furthermore, the transversion mutation T26248G in exon 7 of *NSUN7* altered protein folding and led to a reduction in sperm motility in asthenospermic men [127].

#### Autoimmune diseases

Systemic lupus erythematosus (SLE) is a complex autoimmune disorder characterized by the loss of normal CD4^+^ T cell activity balance. Using chromatography-coupled triple quadrupole mass spectrometry, Guo et al. studied 11 methylation modifications, including m^5^C, and discovered that CD4^+^ T cells from SLE patients had lower m^5^C levels but more m^5^C-containing mRNAs than CD4^+^ T cells from healthy controls. Subsequent RNA-BisSeq and bioinformatics analysis revealed that the majority of hypermethylated or up-regulated genes in SLE were involved in immune-related and inflammatory pathways, such as cytokine signaling, interferon signaling, and immune system [128].

#### Colitis

As reported by Yang et al. [129], NSUN2 deficiency in Th17 cells reduced the stability of m^5^C-modified mRNAs such as *Il17a* and *Il17f*, resulting in improved colitis development generated by Th17 cells in a newly described dextran sulfate sodium (DSS)-induced animal colitis model. RORγt could facilitate the binding of NSUN2 to chromatin areas of their targets resulting in transcription-coupled m^5^C production and increase mRNA stability. This study demonstrated that NSUN2 could be a therapeutic target for autoimmune illness [129].

#### Infectious diseases

RNA m^5^C methylation also plays a crucial function in viral infections. NSUN2 has been shown to promote the replication of many viruses, such as human metapneumovirus, respiratory syncytial virus, Sendai virus, vesicular stomatitis virus, and human immunodeficiency virus (HIV), through the m^5^C-dependent pathway [130,131]. In contrast, the HIV-1 restriction factor *NSUN1* was reported to interact with HIV-1 transactivation response region (*TAR*) RNA through competition with the HIV-1 Tat protein and contribute to m^5^C modification of *TAR*, which inhibited HIV-1 transcription and promoted viral latency [132]. Another m^5^C methyltransferase, DNMT2, was found to promote HIV-1 RNA stability through RNA methylation [133]. In 2020, Eckwahl et al. discovered an unusually high amount of m^5^C in murine leukemia virus genomic RNA, compared with that in uninfected cellular mRNAs. The reader protein ALYREF, which uniquely identifies m^5^C modifications of viral RNA, was also shown to regulate viral production [67]. Based on these findings, the modulation of m^5^C methylation may facilitate the treatment of viral infectious diseases.

In summary, RNA m^5^C methylation is a molecular mechanism that controls the expression of eukaryotic genes important for a wide range of human diseases. The emergence of novel genome-wide sequencing technologies has revealed anomalous m^5^C modifications and their corresponding regulatory proteins in a range of human diseases. Mutations and variations in the expression of genes encoding numerous NSUN proteins have been associated with various human diseases, emphasizing the need for further characterization of this RNA methyltransferase family. In this section, we highlighted recent advances in elucidating the functions of m^5^C alterations and their associated regulators in important disease categories, including hereditary disorders, cancers, infectious diseases, and other pathological conditions, such as infertility and immune diseases (**Table 2**).

**Table 2:** Roles of m^5^C RNA methylation in various diseases

Disease	Category	Protein	Change (disease *vs*. normal)	Function	Molecular mechanism	Related RNA	Ref.
**Hereditary diseases**							
DS	Writer	NSUN2	Deletion	Causal gene	NSUN2-depletion could cause growth retardation, mild microcephaly, and learning disabilities	tRNA^Asp(GTC)^	[98]
Autosomal-recessive intellectual disability	Writer	NSUN2	Deletion	Causal gene	Unknown	tRNA	[96]
	Writer	NSUN2	Mutation	Causal gene	The missense change c.2035G>A (p.Gly679Arg) in NSUN2 could cause it to fail to localize within the nucleolus	m^5^C34 of tRNA^leu(CAA)^	[97]
Neuro-developmental disorders	Writer	NSUN2	Deletion	Causal gene	NSUN2 and m^5^C deficiency resulted in the reduced cell size and increased death of cortical, hippocampal, and striatal neurons	tRNA	[36]
Mitochondrial respiratory chain complex deficiency	Writer	NSUN3	Mutation	Causal gene	NSUN3 was associated with the efficient translation and energy metabolism of the mitochondria	m^5^C34 of mt-tRNA	[44]
Cardiac OFT disorders	Writer	NSUN5	Mutation	Causal gene	Loss of NSUN5 function impaired the m^5^C modification and translation efficiency of essential cardiac genes	*Tpm1*	[104]
WBS	Writer	NSUN5	Microdeletion/deletion	Causal gene	The absence of NSUN5 could result in a decrease in total protein synthesis and normal development	m^5^C3782 of 28S rRNA	[20]
	Writer	NSUN5	Deletion	Causal gene	NSUN5 regulated radial glial scaffolds of radial glial cells to limit migration of neocortical neurons during cerebral cortex development	/	[103]
**Cancers**							
ESCC	Writer	NSUN2	Elevated	Oncogene	NSUN2 methylated NMR which is a key regulator of ESCC tumor metastasis and drug resistance	lncRNA (NMR)	[105]
	Writer	NSUN2	Elevated	Oncogene	NSUN2 enhanced the initiation and progression of ESCC via m^5^C-LIN28B-dependent stabilization of *GRB2* mRNA	*GRB2* mRNA	[106]
UCB	Writer and reader	NSUN2 and YBX1	Elevated	Oncogene	NSUN2 and YBX1 stabilized the mRNA of the oncogene *HDGF* that mediated UCB pathogenesis in human	*HDGF*	[11]
GC	Writer	NSUN2	/	Oncogene	NSUN2 promoted the proliferation, migration, and invasion of gastric cancer cells	*PIK3R1* and *PCYT1A*	[107]
Glioma	Writer	NSUN5	/	Tumor-suppressor characteristics	NSUN5 loss derived an overall depletion of protein synthesis, and led to the emergence of an adaptive translational program for survival under conditions of cellular stress	m^5^C3782 of 28S rRNA	[53]
Glioblastoma	Writer	NSUN5	Up-regulated	Oncogene	NSUN5 promoted tumor growth by facilitating the increased protein synthesis required for tumor progression	m^5^C3782 of 28S rRNA	[54]
Pancreatic cancer	Writer	NSUN6	Decreased	Anti-oncogene	NSUN6 suppressed the proliferation of pancreatic cancer cells	/	[112]
	Writer	NSUN2	Up-regulated	Oncogene	NSUN2 regulated cancer progression and epithelial differentiation	Various RNAs	[110]
Hepatocellular carcinoma	Writer and reader	NSUN2 and G3BP1	Elevated	Oncogene	NSUN2 methylated *H19* gene and thus promoted its stabilization and exerted its oncogenic effect	*H19* lncRNA	[108]
Acute myeloid leukemia	Eraser and reader	TET2 and YBX1	Decreased	Anti-oncogene	TET2 deficiency resulted in the increase of m^5^C in *TSPAN13* mRNA; YBX1 specifically recognized this modification and increased the stability and expression of this transcript	*TSPAN13*	[71]
**Other diseases**							
Infertility	Writer	NSUN7	T26248G-transversion mutation	Causal gene	The mutation caused serine to be converted to alanine, affected the shape of the helix, coil, and strand, and altered protein folding and ligand binding sites	/	[127]
	Writer	NSUN7	A11337-deletion mutation	Causal gene	The mutation resulted in the codon GTA of Val157 replaced with stop codon TAG, causing an abortive protein product with amino acid sequence shorter than normal	/	[126]
Immune system disorders							
SLE	Writer	NSUN2	Decreased	Causal gene	Hypermethylated m^5^C or/and significantly up-regulated genes in SLE were significantly involved in immune-related and inflammatory pathways	/	[128]
Colitis	Writer	NSUN2	/	Causal gene	Deletion of *Nsun2* in mouse CD4^+^ T cells specifically inhibits Th17 cell differentiation and alleviates Th17 cell-induced colitis pathogenesis	*Il17a* and *Il17f*	[129]
Infectious diseases							
hMPV, RSV, SeV, VSV	Writer	NSUN2	/	Promote viral replication	NSUN2 controlled antiviral innate immunity through modulating the m^5^C methylome of ncRNAs and their expression	ncRNAs (RPPH1 and 7SL RNAs)	[130]
HIV-1	Writer	NSUN2	/	Promote viral replication	NSUN2 serves as a post-transcriptional regulator, playing a pivotal role in both the splicing and functionality of HIV-1 mRNA	HIV-1 RNAs	[131]
	Writer	NSUN1	/	HIV-1 restriction factor	NSUN1 interacted with HIV-1 *TAR* RNA by competing with HIV-1 Tat protein and contributed to *TAR* m^5^C methylation	*TAR* RNA	[132]
	Writer	DNMT2	/	Promote viral replication	DNMT2 relocalized from the nucleus to the stress granules and methylated HIV-1 RNA	HIV-1 RNA	[133]
Retrovirus	Reader	ALYREF	/	Promote viral replication	ALYREF promoted retrovirus replication	Retrovirus RNA	[67]

*Note*: DS, Dubowitz-like syndrome; ESCC, esophageal squamous cell carcinoma; HIV, human immunodeficiency virus; hMPV, human metapneumovirus; OFT, outflow tract; RSV, respiratory syncytial virus; SeV, Sendai virus; SLE, systemic lupus erythematosus; UCB, urothelial carcinoma of the bladder; GC, gastric cancer; VSV, vesicular stomatitis virus; WBS, William-Beuren syndrome.

## Potential applications of RNA m^5^C methylation in human diseases

**Figure 4:**
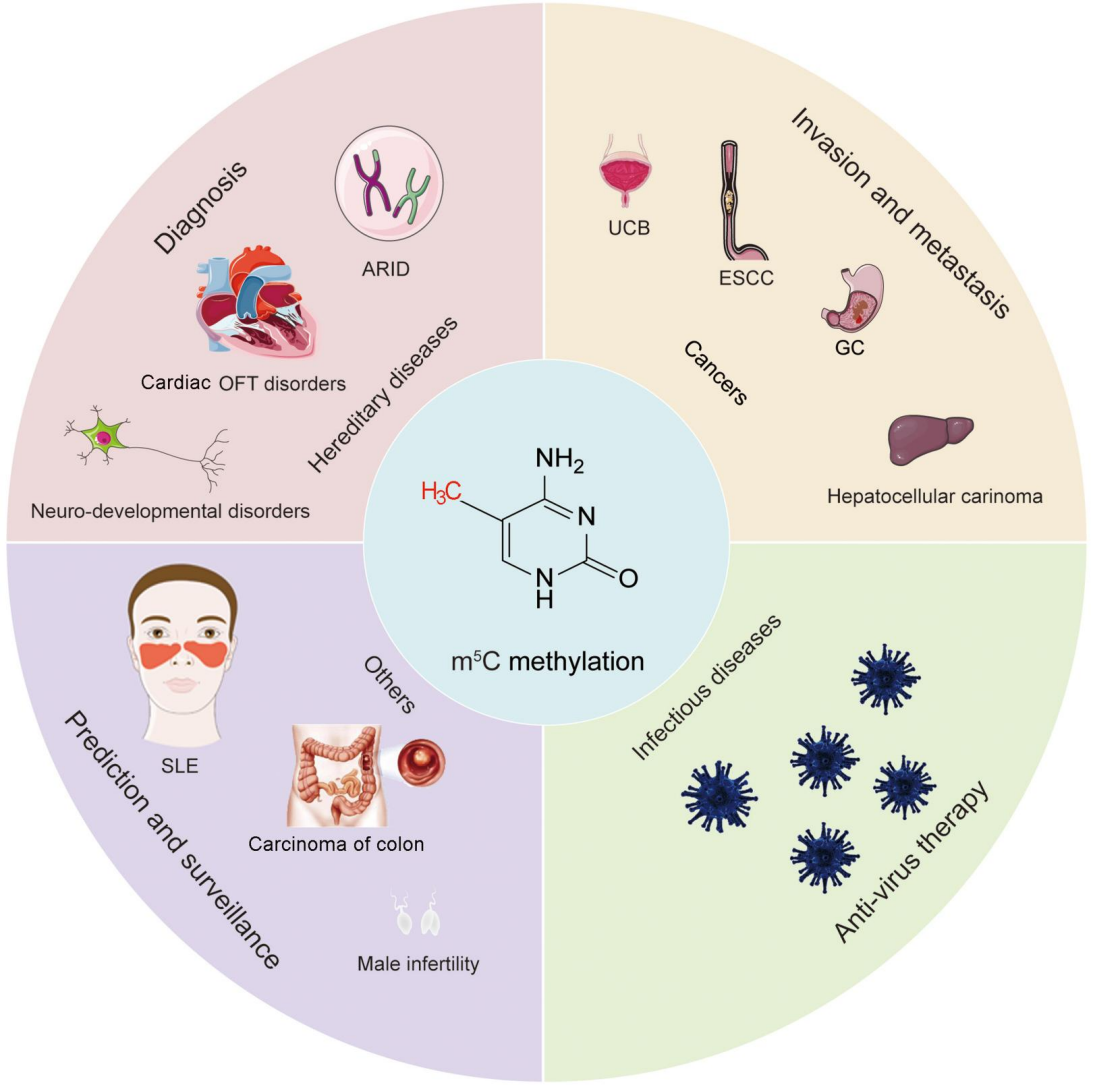
Potential applications of RNA m^5^C methylation OFT, outflow tract; ARID, autosomal-recessive intellectual disability; SLE, systemic lupus erythematosus.

Given that mutations, deletions, or variants in the genes of many NSUN family proteins are associated with a variety of neurological disorders, detecting these changes can facilitate the diagnosis of these neurological disorders. With the advancements in gene therapy, these proteins can also serve as potential targets for disease treatment. Additionally, various regulatory factors of m^5^C methylation have been associated with tumor development, invasion, and metastasis. These regulatory factors can also be employed as biomarkers for cancer prediction and disease progression. Previous studies have revealed several m^5^C methylation transferase enzymes that could influence viral replication. Agonists and inhibitors targeting these enzymes can be explored as therapeutic options for viral infectious diseases. Furthermore, m^5^C methylation modifications have been associated with conditions such as SLE, colitis, and male infertility, offering the potential for disease monitoring in these cases. As research into the etiology of human diseases continues, RNA m^5^C methylation may be employed in the diagnosis, detection, and treatment of a broader spectrum of disorders. In conclusion, we believe that RNA m^5^C methylation may have significant potential in the diagnosis, prognosis, and treatment of human diseases. **Figure 4** shows the potential applications of RNA m^5^C methylation.

## Final remarks and the outlook

The role of epigenetic RNA changes in chromatin remodeling and gene expression is becoming increasingly evident. RNA m^5^C modifications and their regulators are found in various subcellular organelles, including the cytoplasm, Golgi apparatus, nuclear particles, and mitochondria. Gaining insight into how RNA epigenetics regulates the activities of these subcellular organelles would improve our ability to describe a variety of physiological processes as well as pathological conditions. Despite substantial advances in understanding the physiological significance of RNA epigenetics, the precise mechanisms through which RNA loci are altered and how regulators of epigenetic modifications influence disease progression remain largely unknown. The development of reliable methods for detecting m^5^C modifications is critical for understanding the regulation of RNA properties. Moreover, whether m^5^C modifications co-regulate gene expression in cooperation with other methylation modifications, such as m^6^A, 1‐methyladenosine (m^1^A), and *N*^7^-methylguanosine (m^7^G), remains unknown. The question of the intrinsic link between these methylation modifications should be explored in future studies.

In recent years, although much progress has been made in understanding the mechanisms of RNA m^5^C methylation through the development of various methods such as MeRIP-seq, RNA-BisSeq, and nanopore sequencing, there remain several unresolved questions. First, studies on RNA m^5^C modification have mainly focused on RNA methyltransferases, and little is known about m^5^C erasers for various RNA species. Therefore, it is important to identify novel m^5^C readers and erasers to understand the mechanisms underlying various human diseases. Second, RNA alterations and its associated regulators have a high potential for use as diagnostic and prognostic tools, as well as therapeutic intervention targets. However, given the importance of RNA m^5^C modifications, much work remains to be done to fully understand their pathophysiological significance and the roles of associated regulators in human diseases. Therefore, it is critical to create animal models with knockout of methylation-related proteins to investigate their precise functions, which has not been the focus of previous studies. Third, there are few specific activators and inhibitors of proteins mediating m^5^C modification, which limits the options available for treatment of human diseases caused by disturbances in epigenetic methylation marks. Therefore, the development of specific methyltransferase agonists and inhibitors should be a major direction for future research.

## CRediT author statement

**Yanfang Lu:** Writing – original draft, Writing – review & editing, Funding acquisition. **Liu Yang:** Investigation, Visualization. **Qi Feng:** Investigation, Visualization. **Yong Liu:** Investigation, Visualization. **Xiaohui Sun:** Investigation, Visualization. **Dongwei Liu:** Supervision, Writing – review & editing. **Long Qiao:** Supervision, Writing – review & editing, Funding acquisition. **Zhangsuo Liu:** Supervision, Writing – review & editing. All authors have read and approved the final manuscript.

## Competing interests

All authors have declared no competing interests.
